# Generating CRISPR/Cas9-Derived Mutant Mice by Zygote Cytoplasmic Injection Using an Automatic Microinjector

**DOI:** 10.3390/mps1010005

**Published:** 2018-01-12

**Authors:** Brendan Doe, Ellen Brown, Katharina Boroviak

**Affiliations:** Wellcome Trust Sanger Institute, Hinxton, CB10 1SA, UK; es5@sanger.ac.uk (E.B.); kb16@sanger.ac.uk (K.B.)

**Keywords:** cytoplasmic injection, CRISPR/Cas9, microinjection, zygotes

## Abstract

Clustered regularly interspaced short palindromic repeats (CRISPR)/CRISPR-associated (Cas) assisted generation of mutant animals has become the method of choice for the elucidation of gene function in development and disease due to the shortened timelines for generation of a desired mutant, the ease of producing materials in comparison to other methodologies (such as embryonic stem cells, ESCs) and the ability to simultaneously target multiple genes in one injection session. Here we describe a step by step protocol, from preparation of materials through to injection and validation of a cytoplasmic injection, which can be used to generate CRISPR mutants. This can be accomplished from start of injection to completion within 2–4 h with high survival and developmental rates of injected zygotes and offers significant advantages over pronuclear and other previously described methodologies for microinjection.

## 1. Introduction

Since the first publications reporting generation of mutant animals using the CRISPR/Cas system in 2013 [1,2] there has been an explosion in the use of this technology and it has fast changed from being a novel method for introducing targeted genomic sequence changes in mice, to a routine mainstream methodology. Detailed protocols and publications describing the use of CRISPR/Cas9 as a tool for the generation of targeted double-strand breaks (DSBs) in the genome which can then be repaired via non homologous end joining (NHEJ) or homology-directed repair (HDR) can be found in abundance [3,4,5,6,7,8] but few researchers describe the methodology and protocols for introducing CRISPR/Cas9 into the mouse zygote via micromanipulation into the cytoplasm. Previously described micromanipulation methodologies to introduce CRISPR/Cas9 reagents into the cytoplasm of a one-cell mouse zygote either require a high degree of operator skill or results described show poor embryo survival, low efficiencies or lack detailed description of the methodologies used [9,10,11,12,13]. In some cases, low efficiencies may be down to technical issues with penetrating the very flexible oolemma of the zygote, resulting in the membrane invaginating around the needle tip and non-delivery of DNA constructs or transposons. Injection directly into the pronucleus of a one-cell zygote is an established method for generating mutant mice in traditional overexpression transgenics and has also been used to introduce CRISPR/Cas9 reagents into zygotes [14]. However, some concerns have been raised about the introduction of chromosomal breaks caused by pronuclear injection [15] and in some mouse strains the pronucleus is hard to visualise, or is small adding to the difficulty in delivering endonuclease to the target area. We have also seen that, in comparison with cytoplasmic injection, developmental and birth rates post-injection into the pronucleus are not as good, possibly due to mechanical damage of the sticky nucleolus during the injection process and this observation agrees with other published data [16]. Other published protocols for the generation of CRISPR/Cas9 mutants use piezo-driven micropipettes of comparatively large bore size, a complex drop set up and potential use of toxic chemicals [17]. In addition, this equipment is specialised and requires a high degree of operator skill and may not be readily available in many laboratories. Furthermore, there have been several recent reports of electroporation as a method for introducing CRISPR/Cas into zygotes [18,19]. However, these techniques are not yet widely adopted or validated, but may in the longer term provide another route for generation of genetically modified mice. 

The present microinjection protocol we have developed describes cytoplasmic injection for generation of CRISPR/Cas9 mutants using a constant flow of air pressure from a pressurised injection set up. Cytoplasmic injection may also be successfully used for introduction of transposons, zinc-finger nucleases, TALENs (transcription activator-like effector nucleases) and modified DNA [10,11,12,13,20,21,22]. This injection protocol can be used to rapidly inject embryos, 30 embryos can be injected in 20 min by a fully trained microinjectionist and without the aid of substances such as cytochalasin B used to make the oocyte more fluid during injection and less prone to lysis [23]. Our methodology has been used for the generation of CRISPR/Cas9 mutants with exon deletions in a high-throughput manner (over 150 lines now generated for the International Mouse Phenotyping Consortium, IMPC) [24] as well as generation of large deletions up to 1.15 Mbp [25], point mutations [26] and conditional alleles by loxP insertions. This has been achieved with high survival rates of zygotes post-injection (on average 80% of zygotes surviving with up to 100% survival), good rates of development and birth rates (on average 30% but up to 60% of offspring developing to term) and a high efficiency in mutant generation (on average 65% but up to 100% of offspring born showing endonuclease activity) even in difficult to use inbred strains such as C57Bl/6NTac (see Section 4). These parameters allow measurable benchmarks for beginners to gauge the success of their technique. It has proven challenging to introduce large fragments such as green fluorescent protein (GFP) or *lacZ* β-galactosidase using CRISPR/Cas9-mediated homologous recombination by direct injection into zygotes. Until this aspect of mutant generation with CRISPR has improved significantly it is difficult to predict for these experiments whether injection into the cytoplasm or the pronucleus will give better success rates. 

## 2. Experimental Design

This protocol describes the microinjection of CRISPR/Cas endonucleases into the cytoplasm to allow genome editing at precise locations. A pool of zygotes for microinjection are produced by superovulation and mating with stud males. One-cell embryos are harvested from plugged females, treated with hyaluronidase to remove cumulus cells and washed in preparation for cytoplasmic microinjection. 

Cytoplasmic injection is performed using an inverted microscope with micromanipulators. Microinjection tips are connected to an automatic injector to deliver the CRISPR/Cas materials under positive pressure to the cytoplasm of the zygote. Injected embryos are then briefly cultured to assess viability before being transferred to females made pseudopregnant by mating to vasectomised males and allowed to develop to term. A couple of weeks after birth, pups are ear-clipped to take material for genotyping to look for genetically altered animals and hence assessment of success of the cytoplasmic injection. As a control of injection technique, developmental rates of zygotes and guide RNA (gRNA) cutting efficiency a small portion of injected and non-injected embryos can be left in culture to 3.5 days post coitum d.p.c. blastocysts. This will allow genotyping to be performed on these blastocysts and give a comparison of injected vs. non-injected developmental rates [27].

### 2.1. Materials

- C57BL/6Ntac female mice, age 4–6 weeks, for zygote collection
- F1 (CBA <WTSI>; C57BL/6J-Jax F1), mice for use as pseudopregnant females for embryo transfer post-zygote microinjection and vasectomized males.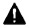 **CAUTION** Experimental procedures involving animals must be carried out according to all relevant institutional and governmental regulations.
- Pregnant mare serum gonadotropin (PMSG; Intervet, Milton Keynes, Buckinghamshire, UK; Cat. no.: Folligon 5000 IU)
- Human chorionic gonadotropin (hCG; Intervet; Cat. no.: Chorulon 1500 IU)
- Potassium-supplemented simplex optimised medium (KSOM medium; AMS Biotechnology (Europe) Limited, Abingdon, Oxfordshire, UK; Cat. no.: GSM 5140)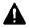 **CRITICAL STEP** Store KSOM medium at −20 °C. After thawing, keep it at 4 °C and use it within 2 weeks. Use a fresh aliquot each day.
- Hyaluronidase (Sigma-Aldrich Company Ltd., Gillingham, Dorset, UK; Cat. no.: H3506)
- M2 medium (Sigma-Aldrich Company Ltd; Cat. no.: M7167)
- Flushing holding medium (FHM Media; AMS Biotechnology (Europe) Limited; Cat. no.: GSM 5130),
- Dulbecco’s phosphate-buffered saline 1× (DPBS 1×; Gibco Life Technologies Europe BV, Bleiswijk, South Holland, Netherlands; Cat. no.: 14190-094).

### 2.2. Equipment

- Inverted microscope with differential interference contrast (DIC)/Hoffman optics (Leica DMI 4000B, Leica Microsytems (UK) Ltd., Milton Keynes, Buckinghamshire, UK; or Zeiss Axiovert 200M, Carl Zeiss Ltd., Cambridge, Cambridgeshire, UK)
- Micromanipulator set (Eppendorf TransferMan 4r, Cell Tram Air; Eppendorf UK, Stevenage, Hertfordshire, UK)
- Microinjector (Eppendorf UK FemtoJet 4i)
- Microcentrifuge (Eppendorf UK 5415D)
- Holding pipette (Vacutip; Eppendorf UK; Cat. no.: 5175 108.000)
- Microinjection pipette (also referred to as microinjection needle or tip; Eppendorf UK, Femtotip Cat. no.: 5242 952.008)
- Microinjection pipette loading tips (microloader tips; Eppendorf UK; Cat. no.: 5242 956.003)
- CO_2_ incubator (New Brunswick Galaxy 48 R; Eppendorf UK)
- Stereomicroscope (Leica M125; Leica Microsytems (UK) Ltd.)
- Cavity Slide for microinjection of zygotes VWR International Ltd., Leighton Buzzard, Bedfordshire, UK; Cat. no.: 631-9475)
- One-well culture dishes (Falcon In Vitro Fertilisation (IVF) dish; Corning GmbH, Wiesbaden, Hesse, Germany; Cat. no.: 353653)
- 60 mm Petri dish, Falcon (60 mm × 15 mm; Corning GmbH, Cat. no.: 353004)
- Two pairs of Forceps Dumont #5 (Interfocus Ltd., Linton, Cambridgeshire, UK; Cat. no.: 91150-20)
- 1 mL Pipette and tips (Gilson, Dunstable, Bedfordshire, UK)

## 3. Procedure

Here we give a detailed protocol for the microinjection of zygotes by cytoplasmic injection and outline details of the techniques around this which are previously described [28].

### 3.1. Zygote Preparation. Time for Completion: 1 h

**Figure 1:**
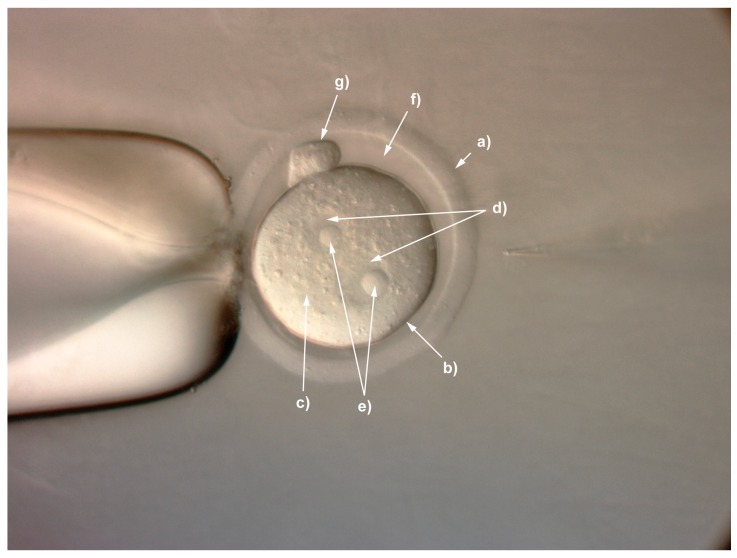
Structures of the fertilized zygote. (**a**) zona pellucida; (**b**) oolemma; (**c**) cytoplasm; (**d**) pronuclei; (**e**) nucleoli; (**f**) perivitelline space; (**g**) polar bodies.

1. Intraperitoneally (IP) inject 10 female C57BL/6NTac (or strain of choice) 4–6 weeks old mice with PMSG (5 IU) at 11.00 a.m.–1 p.m. on day 1.
2. After 48 h (i.e., on day 3), IP inject female mice with hCG (5 IU). After the hCG injection, house female mice with C57BL/6NTac stud male mice overnight for mating.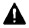 **CRITICAL STEP** The most effective hormone regime to give maximal numbers of eggs will differ between strains and sub-strains and should be determined empirically.
3. 21–22 h post-mating, check female mice for the presence of a copulation plug.
4. Euthanize the mice and collect zygote-cumulus mass complexes by dissecting the oviduct into prewarmed 37 °C PBS ensuring you do not cut the ampulla.
5. Move an oviduct into 1.8 mL of pre-warmed M2 medium and using a stereomicroscope “pop” the oviduct using a pair of forceps at the swollen ampulla region to release the cumulus complex mass. Pin down the oviduct using one pair of forceps whilst gently tearing the ampulla with the other pair. Remove the “popped” oviduct and repeat the process for the remaining oviducts. See **TROUBLESHOOTING, Appendix A (Table A1)**
6. Add 1 vial of thawed hyaluronidase (see Section 5) and gently swirl the dish to disperse cumulus cells. If necessary pipette the masses gently up and down several times with a 1 mL Gilson tip, and wait for the cumulus cells to fall away from the zygotes. This will take 30 s to a couple of minutes.
7. Using a mouth pipette, pick up the embryos and place them into a fresh dish of 500 μL FHM followed by washing the embryos through at least 3 × 100 μL drops of FHM medium.
8. Remove unfertilised embryos and place the remaining fertilised zygotes (see Figure 1 identifying the main structures of the fertilised mouse zygote) into pre-equilibrated KSOM medium at 37 °C in a 5% CO_2_ incubator until ready for injection (see Section 5).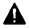 **CRITICAL STEP** Long-term exposure to hyaluronidase can cause embryo degradation. The embryos should remain in hyaluronidase for the minimum time possible. Embryos must be washed through several times in FHM after exposure.

### 3.2. Preparation of CRISPR/Cas9 Materials. Time for Completion: 30 min

9. Prepare the appropriate injection mix depending on the aim of the experiment (Table 1). Dilute the Cas9 mRNA or protein and gRNA in RNase-free water to the working concentration indicated in Table 1. (See also Section 5).
10. Briefly mix all the components and store them at −80 °C until required for microinjection.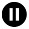 **PAUSE STEP** CRISPR/Cas9 injection mix maybe stored at −80 °C for up to one year without any detrimental effect on activity. However, we do not recommend refreezing materials once thawed for microinjection.

### 3.3. Preparation for Microinjection. Time for Completion: 20 min

**Figure 2:**
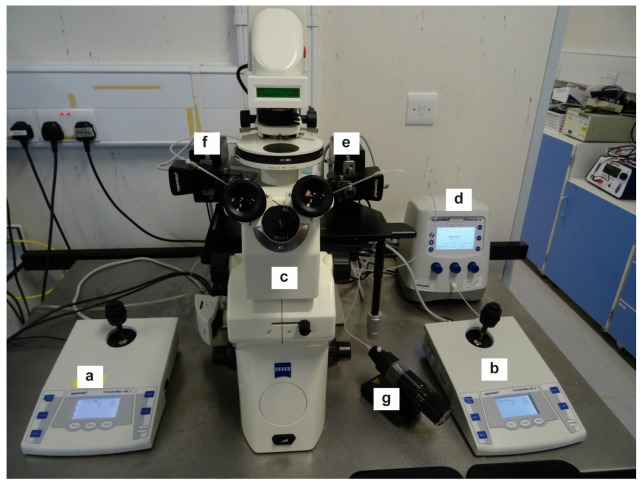
(**a**) Electronic micromanipulator for holding pipette; (**b**) electronic micromanipulator for injection needle; (**c**) inverted microscope; (**d**) Femtojet automatic injector; (**e**) instrument holder for injection needle; (**f**) instrument holder for holding pipette; (**g**) manual microinjector for providing positive and negative pressure to holding pipette.

11. Place 20–30 embryos into a large drop of FHM in the well of a depression slide (100 to 500 μL depending on the size of the depression) or as many as you can comfortably inject in a 30 min period and move to the inverted microscope microinjection rig ready for injection (Figure 2).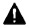 **CRITICAL STEP** Our preference is to not use mineral oil to cover the drop. However, this means that after 30 min or so evaporation of the drop will start to take place and there can be a negative impact on the embryos due to osmotic effects. Change the media between each injection dish and do not place more embryos on the dish than you can inject in a 30 min period. If you start to notice embryos showing signs of osmotic stress such as shrinking or increased lysis rates on injection immediately change to a fresh dish.
12. At 40× times magnification attach the holding pipette to the instrument holder and lower into the cavity slide microinjection dish.
13. Thaw the CRISPR/Cas9 injection mix and spin it for 1 min at 13,200 rpm in the microcentrifuge and store on ice ready for microinjection.
14. Load 2.5–3 μL of injection mix into a Femtotip using a microloader tip. Ensure the mix reaches the tip of the microinjection needle.
15. Attach the Femtotip to the instrument holder connected to the Femtojet and switch on the Femtojet and allow it to reach pressure.
16. Set a balance pressure by adjusting the PC (pressure compensation) range to ca. 40–60 hPa and lower the Femtotip into the microinjection dish until it is the same focal plane as the holding pipette.
17. Increase the magnification to 200–400× and move the Femtotip next to an embryo and ensure both injection tip and zygote are in focus and press ‘Clean’. If the CRISPR/Cas9 injection mix is flowing you should see a visible stream and the embryo will be pushed away by the pressure. See **TROUBLESHOOTING, Appendix A (Table A1)**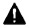 **CRITICAL STEP:** If the pressure compensation is set too high you will have an uncontrolled injection with high volumes of CRISPR materials delivered into the cytoplasm and the embryos will lyse shortly after.

### 3.4. Injection of Zygotes. Time for Completion: 1 h

(See Supplementary Videos 1 and 2).

**Figure 3:**
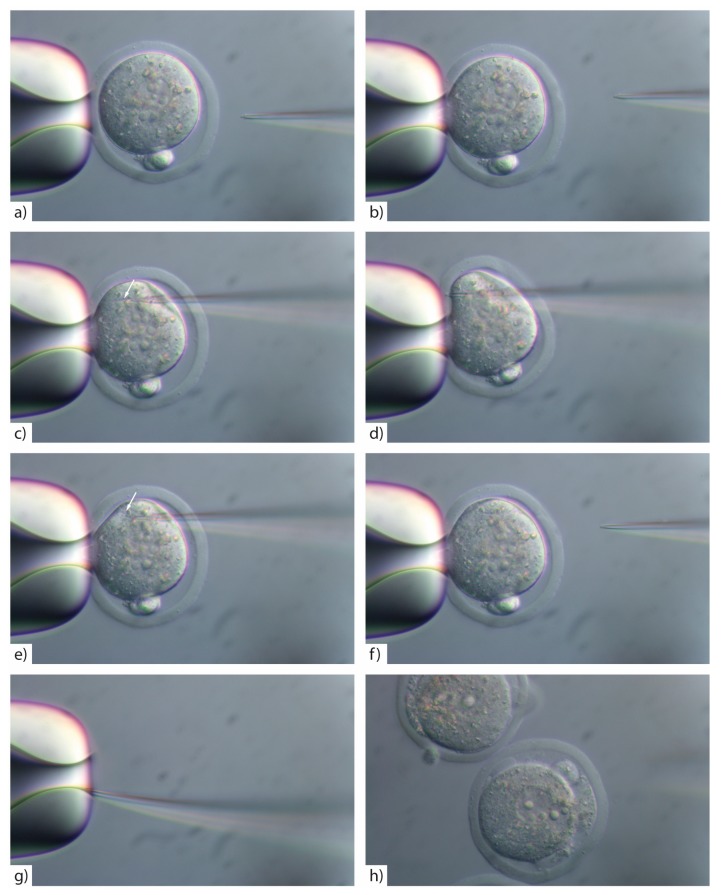
(**a**) Hold the zygote and (**b**) ensure that the cytoplasm is held into the holding pipette (this ensures the embryo will not rotate during the injection process); (**c**) insert the injection pipette into the zygote avoiding the pronucleus and polar bodies and pause briefly halfway inside the egg to see the formation of a small droplet around the injection tip (arrow). This shows the CRISPR/Cas injection mix is flowing; (**d**) push the pipette forward again until it reaches the opposite side of the oolemma, pass the pipette tip through the oolemma to break the membrane and draw back into the embryo; (**e**) look for movement inside the cytoplasm to signify the CRISPR/Cas injection mix has successfully been injected inside the embryo (arrow); (**f**) rapidly withdraw the pipette tip; (**g**) if you need to widen the tip slightly due to clogging, glance the injection pipette tip on the side of the holding pipette to break slightly or inside the holding pipette opening. Adjust down the PC to 15–25 hPa and increase as necessary; (**h**) showing embryos that have lysed post-injection with cytoplasmic content leaking into the perivitelline space.

**Figure 4:**
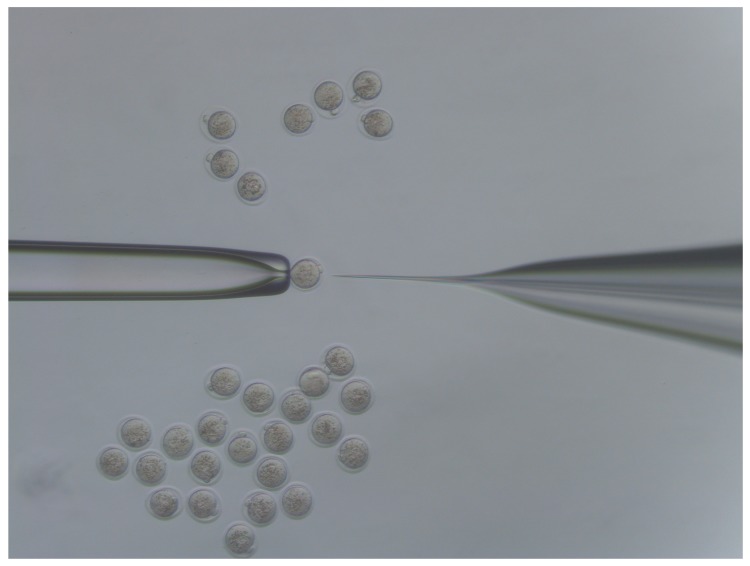
Microinjection dish set up. Dish showing a depression slide filled with flushing holding medium (FHM). Uninjected embryos in the centre of the dish. Injected embryos are moved to the top of the dish. These should be evaluated after injection and lysed embryos removed.

18. Hold the zygote (Figure 3a) ensuring that the cytoplasm is held into the holding pipette so the embryo will not rotate during the injection process (Figure 3b). Also ensure the zygote is held in an orientation where you can avoid the pronucleus and polar body.
19. Insert the injection tip into the zygote and pause briefly halfway inside the egg to see the formation of a small droplet around the injection tip. This shows the CRISPR/Cas injection mix is flowing (Figure 3c). See **TROUBLESHOOTING, Appendix A (Table A1)**
20. Push the pipette forward again until it reaches the opposite side of the oolemma. Pass the pipette tip gently through the oolemma to break the membrane and draw back into the embryo (Figure 3d).
21. Look for movement inside the cytoplasm to signify the CRISPR/Cas injection mix has successfully been injected inside the embryo (Figure 3e). See **TROUBLESHOOTING, Appendix A (Table A1)**
22. Rapidly withdraw the pipette tip (Figure 3f). See **TROUBLESHOOTING, Appendix A (Table A1)**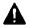 **CRITICAL STEP**: To avoid lysing the zygote, ensure that a minimal amount of CRISPR/Cas mix is injected, by rapidly withdrawing the pipette tip after injection.
23. If the pipette tip becomes blocked with cytoplasmic debris or the mRNA/protein prep contains debris/dust or is sticky due to the oligo prep, widen the tip size slightly by glancing the injection tip on the edge of the holding pipette (Figure 3g).
24. Move injected embryos to the top of the dish and repeat the process for all embryos in the dish (Figure 4).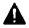 **CRITICAL STEP**: Accurate focus is crucial for several steps in zygote micromanipulation. The injection pipette, zygote and the holding pipette must be in the same horizontal plane.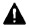 **CRITICAL STEP**: If you widen the microinjection needle tip by glancing off the side of the holding pipette you must reduce the PC as larger volumes of reagents introduced into the cytoplasm will result in embryo lysis (Figure 3h).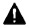 **CRITICAL STEP**: If lysis of embryos is observed after widening the pipette tip and reducing the PC, exchange the injection pipette for a new one.
25. Culture the injected zygotes in KSOM medium at 37 °C in a 5% CO_2_ incubator and 30 min later remove any that might have lysed. The surviving embryos can then be transferred immediately to the oviduct of a pseudopregnant 0.5 d.p.c. recipient or left to culture overnight and transferred the next day to a 0.5 d.p.c. pseudopregnant recipient as two-cell embryos. See **TROUBLESHOOTING, Appendix A (Table A1)**.

### 3.5. Embryo Transfer and Production of Mice. Time for Completion: 3 Weeks

26. Prepare pseudopregnant foster mothers by mating estrus selected F1female mice or chosen strain with vasectomized male mice the day before injection and inspecting for a vaginal plug on the day of injection (0.5 d.p.c.).
27. Perform an embryo transfer of 10–15 one-cell embryos into the oviduct of 0.5 d.p.c. recipients. If you have insufficient recipients to transfer all of your microinjected embryos, culture the embryos overnight and transfer two-cell embryos into the oviduct of 0.5 d.p.c. recipients set up on the day of injection. Recipient mothers deliver pups at approximately 19.5 d.p.c.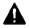 **CRITICAL STEP**: Do not transfer more than 10–15 embryos to each oviduct (20–30/mouse in total). Survival rate of cytoplasmic CRISPR/Cas9-injected embryos can be high so transferring more embryos may result in pregnancy problems for the recipient mother. The vast majority of those embryos that survive microinjection will divide to two-cell embryos (over 90%) so culturing overnight is not advised or necessary.
28. When pups reach two weeks of age ear clip pups and genotype. See **TROUBLESHOOTING, Appendix A (Table A1)**.
29. Genotyped mice can be sexed and weaned at approximately three weeks after birth.

### 3.6. Summarized Time for Completion

- Steps 1–8 Zygote preparation: 1 h
- Steps 9–10 CRISPR/Cas9 preparation: 30 min
- Steps 11–17 Microinjection preparation: 20 min
- Steps 18–25 Injection of zygotes: 1 h
- Steps 26–29 embryo transfer and production of mice: 3 weeks
- Supplemental 1 Design and cloning of tyrosinase gRNAs: 1 week

## 4. Expected Results

After only a few sessions for an experienced microinjectionist survival rates of zygotes post-microinjection should be 75–95% with birth rates on average of 30% of embryos transferred but up to 45–60% commonly achieved. Up to 100% of offspring can show endonuclease activity but this will vary greatly between genes. From 328 CRISPR/Cas projects undertaken at the Wellcome Trust Sanger Institute aiming to delete a critical exon (CE) using cytoplasmic injection of CRISPR/Cas materials, 3776 offspring were born with 65% showing some endonuclease activity and 39% having the desired CE deletion as determined as appropriate by endpoint polymerase chain reaction (PCR), Sanger sequencing and quantitative PCR (qPCR; see Table 2 for results by allele type). Beginners can expect survival rates in the range of 30–50% of embryos injected and much lower mutation rates in offspring due to more embryos remaining uninjected.

## 5. Reagents Setup

- Thaw KSOM and filter through a 0.22 µm filter and aliquot in 5 mL aliquots and keep at 4 °C for up to two weeks.
- Place a single drop of 1 mL of KSOM medium in an IVF one well dish and fill the outer well with 2–3 mL PBS. Do not cover with mineral oil. Then place the dish into a 37 °C incubator preferably overnight or at least 2 h before use to equilibrate
- Thaw FHM and filter through a 0.22 µm filter and aliquot in 5 mL aliquots and keep at 4 °C for up to two weeks.
- Hyaluronidase. From a 100 mg stock powder make a 3 mg/μL working solution by dissolving the powder in 33.33 mL M2 media, filtering through a 0.22 µm filter, aliquoting in 100 μL aliquots in sterile Eppendorf tubes and freezing at −20 °C ready for use.
- PMSG and HCG. Dissolve separately in 0.9% saline to a final concentration of 50 IU/mL and filter through a 0.22 µm filter and aliquot in 1 mL aliquots in sterile Eppendorf tubes and store at −80 °C
- Dilute Cas9 mRNA (Trilink, San Diego, California, USA; Cat. no.: L-7206) or Cas9 protein (LabOmics, Nivelles, Walloon Brabant, Belgium; Cat. no.: Cas9-TOO) to the required working concentration in RNase-free water. Add gRNA (Protocol S1) and if required a single-stranded oligodeoxynuceotide (ssODN) (Table 1)
- ssODN stock, 1 μg/μL (IDT Leuven, Flemish Brabant, Belgium). Resuspend ssODN in RNase-free water to a final concentration of 1 μg/μL. Store the stock at −20 °C.

## Figures and Tables

**Table 1:** Clustered regularly interspaced short palindromic repeats (CRISPR)/CRISPR-associated (Cas) injection mix suggested concentrations.

Aim	Working Concentrations	Components
Indels by NHEJ	Cas9 (50 ng/μL) + gRNA (25 ng/μL)	Cas9 and one gRNA in 30 μL of H_2_O
Point mutation, small tag insertion or insertion of loxP	Cas9 (50 ng/μL) + gRNA (25 ng/μL) + ssODN (100 ng/μL)	Cas9, one (for point mutation) to four (insertion of loxP) gRNAs, ssODN (120–200 bp) in 30 μL of H_2_O
deletion of critical exons or rearrangement of larger fragments	Cas9 (50 ng/μL) + gRNA5a (6.5 ng/μL) + gRNA5b (6.5 ng/μL) + gRNA3a (6.5 ng/μL) + gRNA3b (6.5 ng/μL) + ssODN (100 ng/μL)	Cas9, two to four gRNAs, ssODN (120–200 bp) in 30 μL of H_2_O

NHEJ: non-homologous end joining; gRNA: guide RNA; ssODN: single-stranded oligodeoxynucleotides.

**Table 2:** Mutant production using cytoplasmic injection by allele type.

Desired Allele	No. Genes	No. Embryos Microinjected (MI)	No. Embryos Survived MI (%)	No. Embryos Transferred	No. Embryos Born (%)	Total No. Mutants Born (%) *	No. Mutants with Desired Allele (%)
CE	328	15,613	12,908 (83)	12,304	3776 (31)	2470 (65)	1455 (39)
Point Mutation (SNP)	29	2744	2280 (83)	2140	727 (34)	504 (69)	277 (38) **
Large deletions (9, 376–1, 151, 853 bp)	5	1342	1093 (81)	1063	273 (26)	53 (19) ***	17 (6)

* This includes all mutants where there maybe indels or undesired mutations caused by CRISPR/Cas activity. ** This figure includes mice with SNP only (106) and SNP + Indel (171). *** For large deletions we did not check for indels, so this figure includes only precise and imprecise deletions. CE: Critical exon.

## Supplementary Materials

The following are available online at https://zenodo.org/record/1149611#.Wl83CslObDy, Protocol S1: Design and cloning of gRNAs. Figure S1: Map of the T7-gRNA_ccdB vector used for cloning gRNAs. Figure S2: CRISPR generated Tyr gene mutants. Video S1: Showing Cytoplasmic injection including troubleshooting point 21. Video S2: Showing Cytoplasmic injection including troubleshooting point 21.

## Appendix A

**Table A1:** Troubleshooting guide.

Step	Problem	Possible Reason	Solution
5	Cumulus cells sticks to forceps when removing from oviduct.		Withdraw the forceps from the M2 and surface tension will pull the cumulus encased zygotes into the drop.
17	No injection mix is flowing from the pipette	Cas9 preparation has caused injection needle blockage	Spin injection mix at max speed for 1 min prior to injection
		You have not cleared the injection needle prior to injection	Press ‘Clean’ on the Femtojet to deliver a high pressured burst of air to the needle tip
		Needle is blocked	Gently break off a small piece of the injection needle tip or change needle
19	No droplet is visible around injection tip when tip is in the cytoplasm	Pressure compensation (PC) is insufficient to produce a flow	Increase balance pressure to 40–60 hPa or until you get a flow
		Needle is blocked	Repeat step 17 and press ‘Clean’. If the issue persists gently break off a small piece of the injection needle tip or change needle
21	No movement is visible inside the cytoplasm post-injection	Oolemma has not been broken.	Withdraw the injection needle part way back into the cytoplasm and then reattempt penetration of the membrane at a different point.
		Optics are not focused correctly on cytoplasm	Adjust optics so you can see clearly the needle tip inside the cytoplasm.
	No movement is visible inside the cytoplasm post-injection despite repeated attempts at penetration.	Oolemma has not been broken.	Change needle as it has become blunt.
			Inject the membrane at the point at which the embryo is held by the holding pipette. This point of the membrane is held under negative pressure making penetration easier.
	It’s difficult to control the flow of CRISPR material during injection	PC is too high	Decrease the balance pressure until flow is controlled
		Injection tip size is too large	Change to a fresh needle
22	Cytoplasmic membrane attaches to the needle on exiting embryo post injection	Needle is sticky with cytoplasmic/nucleolar material	Change needle
25	High lysis rate post injection	PC is too high	Reduce balance pressure so that flow of CRISPR/Cas material is delivered in a controlled manner
		Pipette tip is large post breaking the tip	Change needle
		Injection needle is left in the cytoplasm too long	Remove the injection needle rapidly post-injection
		Injection needle and zygote not in the same horizontal plane	Adjust the injection needle so that it is in the same focal plane as the zygote.
		Osmotic effects making zygotes more sensitive to injection	Change to a fresh drop immediately.
	Zygotes appear shrunken prior to injection	Evaporation of media from dish	Change to a fresh drop immediately
28	Low mutant rates	CRISPR materials have not been delivered to the cytoplasm	Ensure the oolemma has been broken and not just invaginated around the pipette.
		CRISPR/Cas9 materials at wrong concentration	Make sure that concentrations are correct and sufficient to generate mutations.
